# Regression of *Schistosoma mansoni* associated morbidity among Ugandan preschool children following praziquantel treatment: A randomised trial

**DOI:** 10.1371/journal.pone.0259338

**Published:** 2021-11-15

**Authors:** Allen Nalugwa, Edridah Muheki Tukahebwa, Annette Olsen, Fred Nuwaha

**Affiliations:** 1 Child Health and Development Centre, College of Health Sciences, Makerere University, Kampala, Uganda; 2 Neglected Tropical Diseases, Vector Control Division, Ministry of Health, Kampala, Uganda; 3 Parasitology and Aquatic Diseases, Faculty of Health and Medical Sciences, University of Copenhagen, Copenhagen, Denmark; 4 Disease Control and Prevention, Makerere University, Kampala, Uganda; International AIDS Vaccine Initiative, UNITED STATES

## Abstract

Preschool children suffer from morbidity attributable to *Schistosoma mansoni*. We compared a single and double dose of praziquantel treatment on the regression of *S*. *mansoni* associated morbidity in children less than six years in Uganda. We measured the sizes of spleen and liver as well as liver fibrosis before treatment and 8 months after treatment among children who either received one dose (n = 201) or two doses (n = 184) of praziquantel (standard oral dose of 40 mg/kg body weight). Heamoglobin measurements were also taken. Overall, liver enlargement reduced from 52.2% (95% CI (Confidence interval) 45.1, 59.3) to 17.9% (95% CI 12.9, 23.9) with a single dose and from 48.4 (95% CI 40.9, 55.8) to 17.9% (95% CI 12.7, 24.3) with a double dose and there was no significant difference between the changes in proportion of children with enlarged liver between the two treatment groups. The proportion of children with enlarged spleen was not significantly reduced in the group treated with either one or two doses, 47.8% (95% CI 41.7, 54.9) to 45.3% (95% CI 38.3, 52.4) and 48.4% (95% CI 40.9,55.8) to 40.8% 95% CI 33.6, 48.2), respectively. Liver fibrosis detected among children getting single dose (n = 9) or double doses (n = 13) resolved after treatment with praziquantel. The number of children with low heamoglobin significantly reduced from 51.2% (95% CI 44.1, 58.3) to 0.5% (0.2, 0.8) and 61.4% (95% CI 53.9,68.5) to 1.1% (95% CI 0.1, 3.9) after single and double dose treatment, respectively. These results suggest that there is no evidence of a difference in effect between one dose of praziquantel and two doses in reversing morbidity attributable to *S*. *mansoni* among children less than six years of age.

## Introduction

Preschool children (PSC) less than six years old get infected [1–5] with *Schistosoma mansoni* and develop associated morbidity such as anaemia and hepatomegaly [6–10]. With heavy infection there is a higher likelihood that the hepatomegaly may progress to hepatic fibrosis even at this early age [6, 8]. Even with these observations, young children less than 94 cm in height (about ≤ 4 years of age) are not included in community and or school-based treatment programmes for schistosomiasis [11]. Several reasons preclude treatment of the young children. First is the ostensive belief that complications of chronic schistosomiasis takes a long time to develop and therefore young children can wait [6, 12]. Second is the lack of child friendly praziquantel for treatment of children [13, 14]. The current tablet of praziquantel is big and bitter with the wherewithal to cause choking and vomiting among young children. Third, determination of the praziquantel dose within this age group using height is a herculean task and is considered rather inaccurate [15]. Finally, because organisation of mass drug delivery for schistosomiasis outside the school system is a logistical nightmare in most of sub-Saharan Africa, pre-school-aged children and out of school children are rarely treated [1, 6, 7]. We and others have previously characterised infection and morbidity associated with *S*. *mansoni* in children less than six years and demonstrated that treatment with praziquantel was safe and efficacious [16–19]. This is a follow-up report to document the long-term effects of such treatment on morbidity associated with intestinal schistosomiasis. In this study, we assessed the impact of praziquantel on *S*. *mansoni* associated morbidity eight months after initial treatment among PSC in Uganda, a country where about one in four people are estimated to be infected with *S*. *mansoni* [2]. This study, therefore, was intended to determine whether *S*. *mansoni*-associated morbidity in PSC regressed after treatment with PZQ, comparing one versus two doses. The information generated is helpful in guiding policy and practice decisions regarding the routine treatment of this important age group as the goal of schistosomiasis control moves toward elimination [20].

## Materials and methods

### Study area and participants

This paper is a continuation of a previous clinical trial, details of study methods described in these reports [3, 9, 17] but will be summarized here. The study was carried out in 26 *S*. *mansoni* endemic fishing communities along Lake Victoria shoreline in eastern Uganda in the districts of Bugiri, Buikwe, Jinja, Mayuge and Namayingo. Children aged 12 to 60 months, who were tested egg positive, were treated with either a single or double dose of PZQ, and were further investigated for *S*. *mansoni* associated morbidity parameters before (June 2013) and 8 months after (February, 2014) chemotherapy. This study is registered with ClinicalTrial.gov.

### Measures

The study procedures included stool parasitological examination, anaemia assessment and abdominal ultrasound examination as described below.

#### Parasitological examination

Following community sensitization on the ongoing study and written consent, caregivers whose children were to participate in the study were given orientation on how to handle and submit the stool samples of their children. Stool containers (polythene sheets) labelled with the child’s identification number and name were given out to the respective parents. For each child one stool sample was collected on three consecutive days; multiple stool collections were proposed due to day-to-day variation in egg counts of *S*. *mansoni* [21, 22]. The Kato–Katz technique was used to prepare stool smears on slides for microscopic examination [23]. Two slides were prepared and examined for each sample; totalling six slides for each child. A small amount of faeces was pressed through a steel screen to remove large debris, the sieved stool filled into a 41.7 mg hole in a template placed on a slide. The specimen on the slide was covered by a piece of cellophane soaked in glycerol with malachite green used as a cover slip. The two faecal smears were each examined under a microscope (100x magnification) and eggs on each slide were counted and recorded by two different experienced field technicians. To ensure the accuracy of the egg counts a 10% of the slides from each field technician were chosen at random and re-read by a senior technician. In case of discrepancies, the slides were re-read and consensus obtained. The outcome infection intensities were classified according to WHO guidelines [24] as light: 1-99EPG, moderate:100-399EPG and heavy: ≥400EPG. Intensity of infection (expressed in eggs per gram of stool, EPG) was calculated by multiplying the mean for the six slides (two slides for each of the three stool samples) by a factor of 24 [25].

#### Anaemia assessment

A finger-prick blood sample was taken from all the recruited children, and their heamoglobin (Hb) concentration (g/dL) was measured using a portable HemoCue^®^ photometer (Ängelholm, Sweden). Anaemia was defined as absent (Hb = >11 g/dL), mild (Hb = 8–10.9 g/dL), moderate (Hb = 6–7.9 g/dL) or severe (Hb<6 g/dL) according to the World Health Organization guidelines [26].

#### Abdominal ultrasound examination

Abdominal ultrasonography was performed using a portable ultrasonographical device (Aloka, SonocameraSSD-500 Tokyo, Japan) with a convex 3.5 MHz transducer, according to WHO standard guidelines [27]. The children were examined lying on their backs with their legs stretched on an examination table. Measurements from the upper to the caudal margin in the left parasternal line (PSL) were done for the spleen length (SL) and the left liver lobe. The size of the right liver lobe was measured in the right mid-clavicular line (MCL), whereas the portal-vein diameter (PVD) was measured at the point where the portal vein enters the porta hepatica at the lower end of the caudate lobe. Height-related reference data from a healthy Senegalese community [27, 28] were used to measure liver and spleen size. The values for organ sizes:, spleen length, left liver lobe (PSL) and right liver lobe (MCL) were then classified as ‘normal’ if they were below or equal to the mean + 2 SD; ‘moderately enlarged’ if they were more than 2 SD but below or equal to the mean + 4 SD, and ‘severely enlarged’ if they were above the mean + 4 SD. In children with texture patterns suggestive of periportal fibrosis (PPF), a picture was taken and the corresponding texture pattern score recorded. Liver texture patterns A and B were considered to show no liver fibrosis while C, D, E, and F indicated disease. In addition, the age, sex, and district were recorded.

### Treatment

The infected children were individually randomized (by an independent statistician) to single or double dose PZQ treatment groups by computer random number generation in Stata/IC 12.0. Each child infected with *S*. *mansoni* was treated according to their weight with PZQ (40 mg/kg body weight) based on standard protocols to ensure adherence and mitigation of side effects [17–19]. The tablets were crushed [16] and mixed with drinking water to facilitate oral uptake in small children [29]. The children were given a piece of bread before drug administration, and orange juice was provided after drug administration to minimize gastrointestinal side effects [13, 30, 31] and mask the sour taste of PZQ [32], respectively. Treatment was performed by an experienced nurse in the presence of each child’s caretaker. The children were also each given a tablet of albendazole (Alzental 400 mg). Two weeks after the first treatment, children who were randomized into the double dose group were given a second dose of PZQ. Treated children remained under supervision for a period of 30 minutes to monitor any immediate reactions as a result of drug administration and the caregivers were further encouraged to report any treatment-related symptoms observed in the children 24 hours post-treatment. In case of adverse events, the affected children would be referred to the nearest local health services.

#### Sample size

The study compared the effect of two different PZQ treatment strategies (single dose versus double dose) on morbidity regression. It was expected that 8 months after treatment, morbidity as measured by liver size would regress by a difference of at least 12% in the two groups (75% in the two-dose group and about 63% in the one dose group). Using the formula of comparison of two proportions [21] with 90% power and at the 5% level of significance the sample size calculated was at least 343 children in each group after adjustment for a 25% loss to follow-up.

### Data analysis

Statistical analysis was carried out in Stata 14 (StataCorp; College Station, TX, USA) in conjunction with SPSS 23 (IBM, New York). To detect a change between follow up and baseline variables, categorical data were compared using the McNemar-Bowker test, and Paired *t*-test was used to compare continuous variables. Statistical significance was defined by a *P* value less than 0.05. To test whether the double dose was different from the single dose in influencing morbidity indices, we compared for a change in parameters before treatment and at follow up within the two groups using the Chi-square test statistic with Yates correction.

### Ethical review

Ethical approval and clearance were obtained from Makerere University, Ethics Committee and the Uganda National Council for Science and Technology (Ref. No. UNCST-HS1274), respectively. The trial was registered on May 30, 2013 with ClinicalTrial.gov, identifier: NCT01901484. The aim and procedure of the study were explained to the caregivers of the recruited children in the local language (Lusoga), and assent obtained before PZQ chemotherapy. Only those children who had written informed consent from their caregivers were included in this study.

## Results

**Fig 1:**
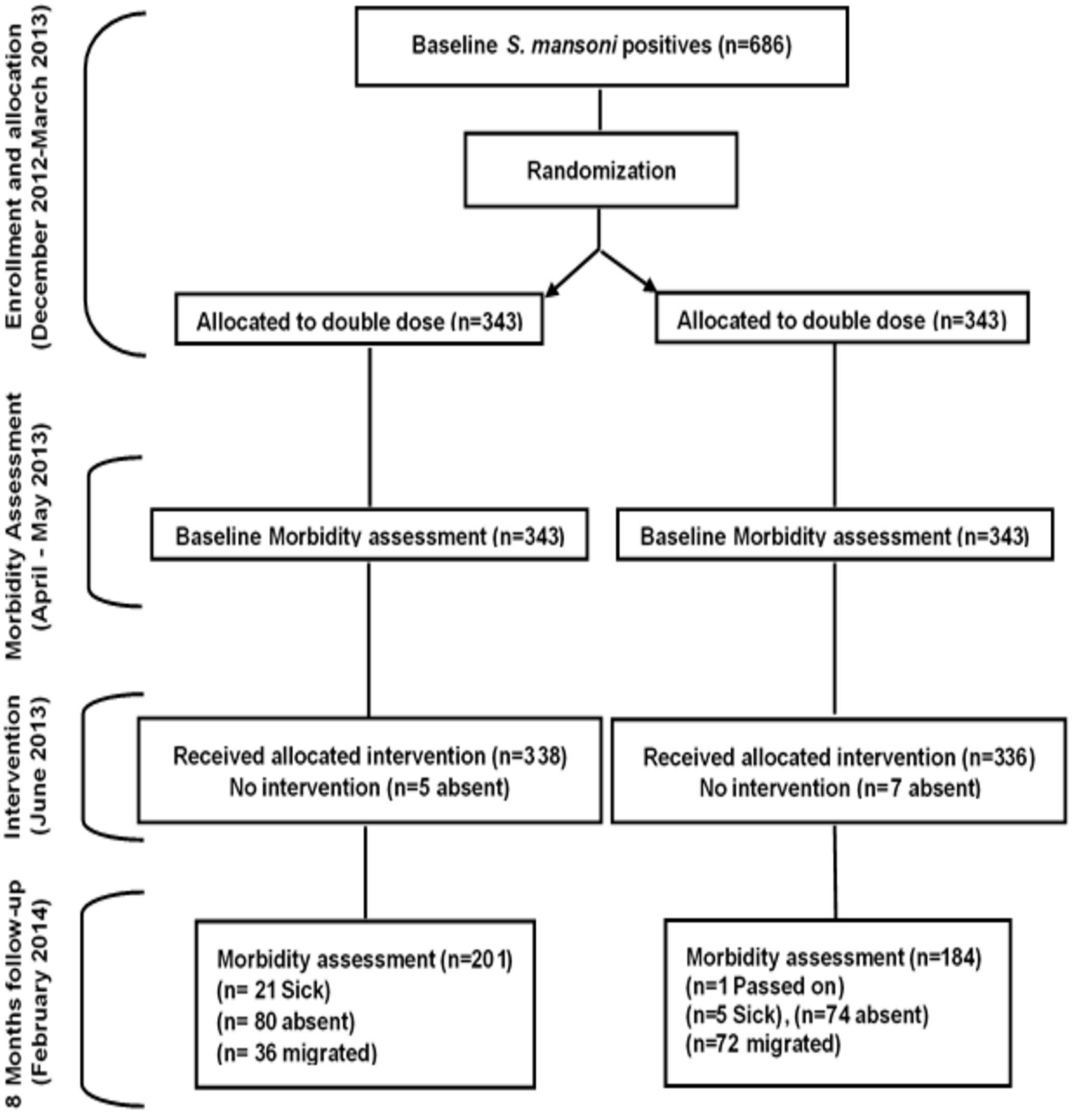
Enrolment, randomization, follow-up, and inclusion in the final analysis comparing between two treatment groups.

Of a total of 686 children who received treatment, 385 were successfully retraced at the 8 months follow-up (201 in the single dose group and 184 in the double dose group) and only these were included in further analysis (Fig 1).

The children who received one dose were similar to the children who received double dose in terms of age sex and infection intensity (Table 1), at eight months follow-up.

**Table 1:** Comparison of preschool children at 8 months (N = 385) with regard to type of treatment, age, sex and infection intensity.

Variable	One Dose n (%) 201 (100)	Two doses n (%) 184 (100)
**Sex**		
Female	111(55.2)	80 (43.5)
Male	90 (44.8)	104 (56.5)
**Age in months**		
≥ 24	7 (3.5)	4 (2.2)
25–36	42 (21)	36 (19.6)
37–48	61 (30.3)	57 (31)
49–60	91 (45.2)	87 (47.2)
**Intensity of infection baseline (epg)**		
1–99	121 (60.2)	103 (56)
100–399	47 (23.4)	40 (21.7)
≥ 400	33 (16.4)	41 (22.3)

The children lost to follow-up (301) and those retained (385) in the study were comparable in terms of age, sex and intensity of infection (Table 2).

**Table 2:** Comparison of preschool children lost to 8 months follow-up (n = 301) and those that were retained in the study (n = 385) with regard to age, sex and infection intensity.

Characteristic	Lost to follow-up n (%) 301 (100)	Retained in study n (%) 385 (100)
**Age group (months)**		
12–24	13 (4.3)	13 (3.4)
25–36	54 (17.9)	75 (19.5)
37–48	92 (30.6)	118 (30.6)
49–60	142 (47.2)	179 (46.5)
**Sex**		
Female	153 (50.8)	191 (49.6)
Male	148 (49.2)	194 (50.4)
**Infection intensity (EPG)**		
Low/light (1–99)	176 (58.5)	224 (58.2)
Moderate (100–399)	70 (23.3)	87 (22.6)
Heavy (≥ 400)	55 (18.2)	74 (19.2)

### Morbidity regression

Morbidity reported here refers to anaemia, liver and spleen sizes as well a liver image patterns including progressive hepatic fibrosis. All children had normal sizes of hepatic portal vein at baseline and at follow-up and the results of portal vein diameter (PVD) are therefore not reported. Table 3 summarizes sonographic observations of morbidity regression detected after treatment with the two different PZQ doses at the two time points; baseline and 8 months post treatment.

**Table 3:** Liver and spleen size between single and double dose treatments before and after treatment with praziquantel.

	Single dose (n = 201)			Double dose (n = 184)		
Characteristic	Pre-treatment	Post-treatment	Pre vs Post treatment	Pre- treatment	Post- treatment	Pre vs Post treatment
	n (%)	n (%)	*P*- value	n (%)	n (%)	*P*- value
**Spleen length (SL)**						
Normal (≤ +2SD)	105 (52.3)	110 (54.7)	ns	95 (51.6)	109 (59.2)	ns
Moderate enlargement (> +2 ≤ +4SD)	63 (31.3)	62 (30.9)		60 (32.6)	46 (25)	
Severe enlargement (≥+4)	33 (16.4)	29 (14.4)		29 (15.8)	29 (15.8)	
**Liver PSL**						
Normal (≤ +2SD)	96 (47.8)	165 (82.1)	<0.001	95 (51.6)	151 (82.1)	< 0.001
Moderate enlargement (> +2 ≤ +4SD)	83 (41.3)	35 (17.4)		73 (39.7)	30 (16.3)	
Much enlarged (≥+4)	22 (10.9)	1 (0.5)		16 (8.7)	3 (1.6)	
**Liver MCL**						
Normal (≤ +2SD)	201 (100)	201 (100)	ns	183 (99.5)	184 (100)	ns
Moderately shrunk (> +2 ≤ +4SD)	0 (0)	0 (0)		1 (0.5)	0 (0)	
Severely shrunk (≥+4)	0 (0)	0 (0)		0 (0)	0 (0)	

ns: not significant at P ≥ 0.05 SD-Standard deviation.

The percent of children with normal spleen (P = 0.87) and liver (P = 0.24) at baseline were similar for those who received single dose compared to those who received double dose.

The proportion of children with enlarged spleen, presented as splenic length, was not significantly reduced in any of the two PZQ treatment groups as the percentage changed from 47.8% (95% CI 41.7, 54.9) to 45.3% (95% CI 38.3, 52.4) in the single dose group and from 48.4% (95% CI 40.9,55.8) to 40.8% 95% CI 33.6, 48.2), in the double dose group. The much-enlarged liver lobe measured as PSL (left parasternal line) significantly (*P* < 0.005) reduced from 10.9% to 0.5% and from 8.7% to 1.6% after single and double dose treatment, respectively. Overall, liver enlargement reduced from 52.2% (95% CI 45.1, 59.3) to 17.9% (95% CI 12.9,23.9) with a single dose and from 48.4 (95% CI 40.9, 55.8) to 17.9% (95% CI 12.7, 24.3) with a double dose. There was no significant change in the right liver lobe size among children treated with single dose compared to those treated with a double dose. The change in liver and spleen sizes were the same among children treated with single dose and those treated with a double dose (*P* > 0.05).

### Liver image patterns and progressive hepatic fibrosis

Liver texture patterns B1, B2, C1 and C2 presented in some children at baseline were completely resolved by both treatments at the 8 months follow up (Table 4).

**Table 4:** Comparison of liver texture pattern between single and double dose before and after treatment with praziquantel.

Characteristic	Single Dose		Double Dose	
	Pre-treatment-n (%)	Post-treatment-n (%)	Pre-treatment- n (%)	Post-treatment-n (%)
**Liver texture pattern (LTP)**				
Normal (A)	183 (91)	198 (98.5)	159 (86.4)	183 (99.5)
Feather streaks (B)	9 (4.5)	3 (1.5)	12 (6.5)	1 (0.5)
Flying saucers (B1)	4 (2.0)	0	5 (2.7)	0
Spider thickening (B2)	2 (1.0)	0	8 (4.4)	0
Peripheral rings (C1)	1 (0.5)	0	0	0
Prominent pipe stems (C2)	2 (1.0)	0	0	0

There was no difference between single dose and double dose regarding normalisation of liver texture patterns (*P* = 0.30).

### Anaemia

At 8 months follow-up there was a significant increase (P < 0.001) in normal Hb values as shown in Table 5. Proportion of children with normal Hb increased from 51.2% (95% CI 44.1, 58.3) at baseline to 95.5% (95% CI 91.7, 97.9) in the single dose group and from 38.6% (95% CI 31.5, 45.6) to 98.9% (95% CI 96.1, 99.9) in the double dose group. The percent mean increases in haemoglobin (follow-up minus baseline Hb divided by baseline Hb) was the same among children who received one dose compared to those who received two doses (One-way ANOVA, F-ratio, 1.037, one degree of freedom, *P* = 0.9).

**Table 5:** Comparison of heamoglobin between single and double dose treatments before and after treatment with praziquantel.

Haemoglobin (g/dL)	Single dose			Double dose		
	Pre-treatment	Post-treatment	p-value	Pre-treatment	Post-treatment	p-value
Normal (>11)	98 (48.8)	192 (95.5)	<0.001	71 (38.6)	182 (98.9)	<0.001
Mild anaemic (8–10.9)	46 (22.9)	5 (2.5)		53 (28.8)	1 (0.5)	
Moderate anaemic (6–7.9)	54(26.9)	4 (2.0)		54 (29.4)	0 (0)	
Severe anaemic (< 6)	3 (1.4)	0 (0)		6 (3.2)	1 (0.5)	

## Discussion

The study compared the effectiveness of a single contrasted with a double dose of PZQ in reversing *S*. *mansoni* associated morbidity in children aged 1–5 years and it clearly shows that a single dose was as effective as a double dose in reduction of the left size of the liver lobe, normalisation of liver image patterns, regression of peri-portal fibrosis and in increasing haemoglobin. Neither single nor double dose PZQ were effective in reducing the size of the spleen in this population.

To our knowledge, this is the first study to follow-up children less than six years infected with *S*. *mansoni* and treated with PZQ for up to 8 months. In a study in Kenya among children follow-up period was up to 4 months and the only morbidity parameter that improved was the proportion of children without anaemia [6, 7]. Splenomegaly, left lobe hepatomegaly and children with image patterns of type B [IPB] increased instead. Possible reasons for these differences compared to our study could be due to: a much higher cure rate attributable to PZQ in our study compared to the Kenyan study [6, 7, 16], increased in malaria prevalence observed in the Kenyan study between baseline and follow-up [6, 7], and differential restricted classification of hepatic pathology into normal versus IPB as well as relatively shorter time of follow-up [33–35].

Our results are however similar to what was observed in Zambia [35] and the Sudan [33] among older children where the proportion of children with grossly enlarged livers significantly reduced. In Zambia the reduction was from 18% to 6.8% 14 months after treatment and in Sudan the reduction was from 27% to 4.6% 23 months after anti-schistosomal therapy. All the pre-school children in our study with abnormal liver texture patterns including those with progressive hepatic fibrosis [image pattern C] reverted to normal after treatment with PZQ. Though the numbers are small these results of our study could suggest that treatment at an early age has a higher likelihood of reversing liver abnormalities and has the wherewithal of completely halting progression of liver pathology associated with *S*. *mansoni* [36]. The fact that liver rather than splenic size reduced after treatment with PZQ indicates that *S*. *mansoni* infections could be responsible for much of the liver morbidity observed in preschool children in this study. We had earlier observed that the size of the left liver lobe, but not the right liver lobe and the spleen, are correlated with intensity of infection with *S*. *mansoni* among children aged less than six years [9]; Other studies [6] also indicate that enlargement of the left liver lobe but not the spleen, is a pattern related to infection with *S*. *mansoni* in children less than six years as well as in older children [33–35].

There was no change in the sizes of the right lobe of the liver as well as of the spleen. All the preschool children included in this study presented with normal right lobe of the liver at baseline and through the eight months follow-up. On the other hand, although over 45% of the children presented with abnormally enlarged spleens at baseline, this did not change following PZQ treatment. The persistent spleen enlargement observed in our study and what has been reported in other studies among preschool children [6] as well as school going children after treatment with PZQ may be attributed to other causes other than *S*. *mansoni* e.g. malaria infection. Malaria is a leading cause of morbidity in children in sub Saharan Africa [37] and spleen enlargement is associated with intensity of transmission [38]. Moreover, malaria and intestinal schistosomiasis are co-endemic in SSA [39] and both have pathological effects on the spleen [40]. The marked increase in Hb after treatment in this study is difficult to interpret. The likely explanation is that anaemia in this study was related with *S*. *mansoni* infections [17] and if so, we can say that decrease in the infections at follow-up led to increased Hb as previously observed elsewhere [10]. However, 9 children (2.3%), who were found severely anaemic, were given iron tablets on top of the PZQ treatment and this may contribute slightly to the increase in Hb. Other common causes of anaemia in Uganda include infections with hookworm and malaria. Malaria prevalence was not assessed in this study and all children in the study were given albendazole although less than 5% were infected with hookworm [3, 17]. Thus, the effects of albendazole and iron treatment could not solely explain the increase in heamoglobin.

## Conclusions

We conclude that hepatomegaly, anaemia as well as liver fibrosis in preschool children infected with *S*. *mansoni* regressed after PZQ chemotherapy. There was no difference between single dose and double dose PZQ in reduction of the *S*. *mansoni* associated morbidity. Thus, our data suggest that preschool children benefit extensively from treatment and should be targeted for treatment in schistosomiasis control programmes. It is recommended that treatment programmes should adopt the use of one dose PZQ, which is equally effective as two doses in reversing schistosomiasis related morbidity. Administration of one dose is more cost-effective than two doses and in addition, it is more operationally feasible.

### Limitations of the study

The loss to follow up was high but this was expected in schistosomiasis endemic regions where migration is very common [41]. Nevertheless, we had enough numbers at 8 months to give us adequate power to (> 0.8) compare changes in liver size assuming a difference of 12% for single dose compared to double dose. Besides, this study compared parameters of the same children at baseline and at follow-up. Therefore, the conclusions of the study still remain relevant as children lost to follow-up are not included in estimating comparator parameters at baseline. A second limitation of the study is that infection with malaria was not assessed. Malaria can modify morbidity associated with *S*. *mansoni*. Although malaria transmission in the study area is perennial and stable with about 40% of children less than five years infected, seasonal spikes occasionally occur [42]. Finally, for ethical reasons it was not possible to have a control group of children that could not be treated.

## Supporting information

S1 File Data file. (SAV)

S2 File Research registration clearance and protocol. (RAR)

## References

[1] Ekpo UF, Oluwole AS, Abe EM, Etta HE, Olamiju F, Mafiana CF. Schistosomiasis in infants and pre-school-aged children in sub-Saharan Africa: implication for control. Parasitology. 2012;139(7):835. doi: 10.1017/S0031182012000029 22313588

[2] Exum NG, Kibira SP, Ssenyonga R, Nobili J, Shannon AK, Ssempebwa JC, et al. The prevalence of schistosomiasis in Uganda: A nationally representative population estimate to inform control programs and water and sanitation interventions. PLoS neglected tropical diseases. 2019;13(8):e0007617. doi: 10.1371/journal.pntd.0007617 31412023 PMC6709927

[3] Nalugwa A, Olsen A, Tukahebwa M, Nuwaha F. Intestinal schistosomiasis among preschool children along the shores of Lake Victoria in Uganda. Acta tropica. 2015;142:115–21. doi: 10.1016/j.actatropica.2014.11.014 25454166

[4] Poole H, Terlouw DJ, Naunje A, Mzembe K, Stanton M, Betson M, et al. Schistosomiasis in pre-school-age children and their mothers in Chikhwawa district, Malawi with notes on characterization of schistosomes and snails. Parasites & vectors. 2014;7(1):153. doi: 10.1186/1756-3305-7-153 24690282 PMC4230191

[5] Seto EY, Sousa-Figueiredo JC, Betson M, Byalero C, Kabatereine NB, Stothard JR. Patterns of intestinal schistosomiasis among mothers and young children from Lake Albert, Uganda: water contact and social networks inferred from wearable global positioning system dataloggers. Geospatial Health. 2012;7(1):1–13. doi: 10.4081/gh.2012.99 23242675

[6] Davis SM, Wiegand RE, Mulama F, Kareko EI, Harris R, Ochola E, et al. Morbidity associated with schistosomiasis before and after treatment in young children in Rusinga Island, western Kenya. The American Journal of Tropical Medicine and Hygiene. 2015;92(5):952–8. doi: 10.4269/ajtmh.14-0346 25758651 PMC4426584

[7] Green HK, Sousa-Figueiredo JC, Basanez M-G, Betson M, Kabatereine NB, Fenwick A, et al. Anaemia in Ugandan preschool-aged children: the relative contribution of intestinal parasites and malaria. Parasitology. 2011;138(12):1534–45. doi: 10.1017/S0031182011001016 21819635

[8] Meurs L, Mbow M, Vereecken K, Menten J, Mboup S, Polman K. Bladder morbidity and hepatic fibrosis in mixed Schistosoma haematobium and S. mansoni infections: a population-wide study in Northern Senegal. PLoS Negl Trop Dis. 2012;6(9):e1829. doi: 10.1371/journal.pntd.0001829 23029589 PMC3459828

[9] Nalugwa A, Nuwaha F, Tukahebwa EM, Olsen A. Schistosoma mansoni-associated morbidity among preschool-aged children along the shores of Lake Victoria in Uganda. Tropical medicine and infectious disease. 2017;2(4):58. 10.3390/tropicalmed2040058 PMC6082064 30270915

[10] Stothard JR, Sousa-Figueiredo JC, Betson M, Bustinduy A, Reinhard-Rupp J. Schistosomiasis in African infants and preschool children: let them now be treated! Trends in parasitology. 2013;29(4):197–205. doi: 10.1016/j.pt.2013.02.001 23465781 PMC3878762

[11] Stothard JR, Sousa-Figueiredo JC, Betson M, Green HK, Seto EY, Garba A, et al. Closing the praziquantel treatment gap: new steps in epidemiological monitoring and control of schistosomiasis in African infants and preschool-aged children. Parasitology. 2011;138(12):1593–606. doi: 10.1017/S0031182011001235 21861945 PMC3178873

[12] Balen J, Stothard JR, Kabatereine NB, Tukahebwa EM, Kazibwe F, Whawell S, et al. Morbidity due to Schistosoma mansoni: an epidemiological assessment of distended abdomen syndrome in Ugandan school children with observations before and 1-year after anthelminthic chemotherapy. Transactions of the Royal Society of Tropical Medicine and Hygiene. 2006;100(11):1039–48. doi: 10.1016/j.trstmh.2005.12.013 16765394

[13] Garba A, Lamine MS, Djibo A, Tahirou A, Aouami MA, Alfari A, et al. Safety and efficacy of praziquantel syrup (Epiquantel^®^) against Schistosoma haematobium and Schistosoma mansoni in preschool-aged children in Niger. Acta tropica. 2013;128(2):318–25. doi: 10.1016/j.actatropica.2012.12.003 23237719

[14] WHO. Report of a meeting to review the results of studies on the treatment of schistosomiasis in preschool-age children. 2011.

[15] Sousa-Figueiredo JC, Betson M, Stothard JR. Treatment of schistosomiasis in African infants and preschool-aged children: downward extension and biometric optimization of the current praziquantel dose pole. International health. 2012;4(2):95–102. doi: 10.1016/j.inhe.2012.03.003 22876272 PMC3407873

[16] Coulibaly JT, N’Gbesso YK, Knopp S, Keiser J, N’Goran EK, Utzinger J. Efficacy and safety of praziquantel in preschool-aged children in an area co-endemic for Schistosoma mansoni and S. haematobium. PLoS Negl Trop Dis. 2012;6(12):e1917. doi: 10.1371/journal.pntd.0001917 23236526 PMC3516585

[17] Nalugwa A, Nuwaha F, Tukahebwa EM, Olsen A. Single versus double dose praziquantel comparison on efficacy and schistosoma mansoni re-infection in preschool-age children in Uganda: a randomized controlled trial. PLoS neglected tropical diseases. 2015;9(5):e0003796. doi: 10.1371/journal.pntd.0003796 26011733 PMC4444284

[18] Namwanje H, Kabatereine NB, Olsen A. The acceptability and safety of praziquantel alone and in combination with mebendazole in the treatment of Schistosoma mansoni and soil-transmitted helminthiasis in children aged 1–4 years in Uganda. Parasitology. 2011;138(12):1586. doi: 10.1017/S0031182011000138 21349218

[19] Sousa-Figueiredo JC, Betson M, Atuhaire A, Arinaitwe M, Navaratnam AM, Kabatereine NB, et al. Performance and safety of praziquantel for treatment of intestinal schistosomiasis in infants and preschool children. PLoS Negl Trop Dis. 2012;6(10):e1864. doi: 10.1371/journal.pntd.0001864 23094120 PMC3475660

[20] Savioli L, Fenwick A, Rollinson D, Albonico M, Ame SM. An achievable goal: control and elimination of schistosomiasis. The Lancet. 2015;386(9995):739. doi: 10.1016/S0140-6736(15)61536-7 26333971

[21] Barakat R, MORSHEDY HE. Efficacy of two praziquantel treatments among primary school children in an area of high Schistosoma mansoni endemicity, Nile Delta, Egypt. Parasitology-Cambridge. 2011;138(4):440. 10.1017/S003118201000154X 21078223

[22] Tukahebwa EM, Magnussen P, Madsen H, Kabatereine NB, Nuwaha F, Wilson S, et al. A very high infection intensity of Schistosoma mansoni in a Ugandan Lake Victoria fishing community is required for association with highly prevalent organ related morbidity. PLoS Negl Trop Dis. 2013;7(7):e2268. doi: 10.1371/journal.pntd.0002268 23936559 PMC3723538

[23] Katz N, Chaves A, Pellegrino J. A simple device for quantitative stool thick-smear technique in schistosomiasis mansoni. Rev Inst Med Trop Sao Paulo. 1972;14(6):397–400. 4675644

[24] WHO. Prevention and control of schistosomiasis and soil-transmitted helminthiasis: report of a WHO expert committee. 2002. 12592987

[25] Mes T, Ploeger H, Terlou M, Kooyman F, Van der Ploeg M, Eysker M. A novel method for the isolation of gastro-intestinal nematode eggs that allows automated analysis of digital images of egg preparations and high throughput screening. Parasitology. 2001;123(3):309–14. doi: 10.1017/s0031182001008496 11578095

[26] WHO. UNU. Iron deficiency anaemia: assessment, prevention, and control. A guide for programme managers, Geneva, WHO. 2001.

[27] Richter J, Hatz C, Campagne G, Bergquist N, Jenkins JM. Ultrasound in schistosomiasis: a practical guide to the standard use of ultrasonography for assessment of schistosomiasis-related morbidity: Second international workshop, October 22–26 1996, Niamey, Niger. Second International Workshop. 2000.

[28] Yazdanpanah Y, Thomas AK, Kardorff R, Talla I, Sow S, Niang M, et al. Organometric investigations of the spleen and liver by ultrasound in Schistosoma mansoni endemic and nonendemic villages in Senegal. The American journal of tropical medicine and hygiene. 1997;57(2):245–9. doi: 10.4269/ajtmh.1997.57.245 9288824

[29] Keiser J, Ingram K, Utzinger J. Antiparasitic drugs for paediatrics: systematic review, formulations, pharmacokinetics, safety, efficacy and implications for control. Parasitology. 2011;138(12):1620–32. doi: 10.1017/S0031182011000023 21349223

[30] Muhumuza S, Olsen A, Katahoire A, Kiragga AN, Nuwaha F. Effectiveness of a pre-treatment snack on the uptake of mass treatment for schistosomiasis in Uganda: a cluster randomized trial. PLoS Med. 2014;11(5):e1001640. doi: 10.1371/journal.pmed.1001640 24824051 PMC4019501

[31] Vennervald BJ, Booth M, Butterworth AE, Kariuki HC, Kadzo H, Ireri E, et al. Regression of hepatosplenomegaly in Kenyan school-aged children after praziquantel treatment and three years of greatly reduced exposure to Schistosoma mansoni. Transactions of the Royal Society of Tropical Medicine and Hygiene. 2005;99(2):150–60. doi: 10.1016/j.trstmh.2004.06.009 15607341

[32] Meyer T, Sekljic H, Fuchs S, Bothe H, Schollmeyer D, Miculka C. Taste, a new incentive to switch to (R)-praziquantel in schistosomiasis treatment. PLoS Negl Trop Dis. 2009;3(1):e357. doi: 10.1371/journal.pntd.0000357 19159015 PMC2614124

[33] Doehring-Schwerdtfeger E, Abdel-Rahim IM, Kardorff R, Kaiser C, Franke D, Schlake J, et al. Ultrasonographical investigation of periportal fibrosis in children with Schistosoma mansoni infection: reversibility of morbidity twenty-three months after treatment with praziquantel. The American journal of tropical medicine and hygiene. 1992;46(4):409–15. doi: 10.4269/ajtmh.1992.46.409 1575287

[34] Mohamed-Ali Q, Doehring-Schwerdtfeger E, Abdel-Rahim IM, Schlake J, Kardorff R, Franke D, et al. Ultrasonographical investigation of periportal fibrosis in children with Schistosoma mansoni infection: reversibility of morbidity seven months after treatment with praziquantel. The American journal of tropical medicine and hygiene. 1991;44(4):444–51. doi: 10.4269/ajtmh.1991.44.444 1904198

[35] Strahan R, McAdam D, Schneider ME. Sonographic response in the liver and urinary bladder of children 14 months after treatment for schistosomiasis. Tropical doctor. 2013;43(2):71–4. doi: 10.1177/0049475513490422 23796675

[36] Homeida M, Defalla A, Kardaman M, Nash T, Fenwick A, Suliman S, et al. Effect of antischistosomal chemotherapy on prevalence of symmers’periportal fibrosis in sudanese villages. The Lancet. 1988;332(8608):437–40. 10.1016/s0140-6736(88)90424-2 2900363

[37] WHO. World malaria report 2019. Geneva: World Health organization; 2019. 2020.

[38] Hay SI, Smith DL, Snow RW. Measuring malaria endemicity from intense to interrupted transmission. The Lancet infectious diseases. 2008;8(6):369–78. doi: 10.1016/S1473-3099(08)70069-0 18387849 PMC2653619

[39] Mbah MLN, Skrip L, Greenhalgh S, Hotez P, Galvani AP. Impact of Schistosoma mansoni on malaria transmission in Sub-Saharan Africa. PLoS Negl Trop Dis. 2014;8(10):e3234. doi: 10.1371/journal.pntd.0003234 25329403 PMC4199517

[40] Booth M, Vennervald BJ, Kenty L, Butterworth AE, Kariuki HC, Kadzo H, et al. Micro-geographical variation in exposure to Schistosoma mansoni and malaria, and exacerbation of splenomegaly in Kenyan school-aged children. BMC Infectious Diseases. 2004;4(1):1–11. 15147584 10.1186/1471-2334-4-13 PMC421731

[41] Abaasa A, Asiki G, Mpendo J, Levin J, Seeley J, Nielsen L, et al. Factors associated with dropout in a long term observational cohort of fishing communities around lake Victoria, Uganda. BMC research notes. 2015;8(1):1–7. 26703879 10.1186/s13104-015-1804-6 PMC4690385

[42] Statistics UBo, International I. Uganda Malaria Indicator Survey 2014–15. UBOS and ICF International Kampala, Uganda, and Rockville, Maryland, USA; 2015.

