# Supplementary material for: Regression of Schistosoma mansoni associated morbidity among Ugandan preschool children following praziquantel treatment: A randomised trial
Source: PLoS One. 2021 Nov 15;16(11):e0259338. doi: 10.1371/journal.pone.0259338 (PMC8592404; doi:10.1371/journal.pone.0259338)
Supplement: Attachment — Submitted filename: Response to Reviewers.docx [file pone.0259338.s003.docx]

**REBUTTAL LETTER**

The Editor

PLOS ONE

We were pleased to have an opportunity to revise our manuscript now entitled **“Regression of *Schistosoma mansoni*-associated morbidity among Ugandan preschool children following praziquantel treatment: A randomized trial”** (PONE-D-20-12223R1).

We have carefully considered the academic editor and reviewer (s) comments and suggestions in the revised manuscript. As instructed, we have attempted to respond to all comments in point-by-point fashion.

Overall, the comments were very helpful and we are truly thankful for the constructive feedback on our original submission. We feel the quality of the manuscript is much improved after addressing the issues raised.

Sincerely,

Allen Nalugwa

(Corresponding Author)

**Academic editor comments:**

i. Please ensure that your manuscript meets PLOS ONE's style requirements, including those for file naming.

**Response: Followed the online PLOSOne formatting sample for guidance.**

ii. Please upload a copy of Supporting Information Figure S2 Fig 1 which you refer to in your text on page 12.

**Response: Supporting Information of Figure S2 Fig 1 which is referred to in the text on page 14 has been uploaded.**

iii. Please provide more details on the methods described under the 'Measures' subsection of your manuscript.

**Response: More detail for assessment of anaemia and examination of infections and abdominal ultrasound is described as advised. Pages 4-7; lines 116 – 178. The treatment with praziquantel is also described further.**

iv. Please provide the original sample size calculation.

**Response: Sample size provided on page 4; lines 108-115.**

v. Thank you for stating in your ethics statement "Only those children who had written informed consent from their caregivers were included."

a**. Response: This has been stated in the ethics review as “Only those children who had written informed consent from their caregivers were included in this study.” Lines 193 – 194.**

b. **The statement** **“The trial was registered with ClinicalTrial.gov, identifier: NCT01901484" is now included in the ethics statement.**

c. **The entire statement/information** "Only those children who had written informed consent from their caregivers were included" and thank you for stating in the text of your manuscript that "Ethical approval and clearance were obtained from Makerere University, Ethics Committee and the Uganda National Council for Science and Technology (Ref. No. UNCST-HS1274), respectively. The trial was registered with ClinicalTrial.gov, identifier: NCT01901484", **has been included in the ethics statement in the online submission form.**

vi. We note that your study was registered on May 30, 2013 at ClinicalTrial.gov. Please add this information to the text of your manuscript.

**Response: This has been stated in Line 190.**

**Reviewer #1:**

i. a. Why are preschool age children not typically included in mass drug administration (MDA) campaigns?

**Until recently, it was thought that younger children don’t develop complications of chronic schistosomiasis and therefore don’t have to be treated. There is lack of child friendly treatment for children; the current praziquantel tablet is big and bitter. Lines 80 - 88.**

b.	Were the children treated with whole or crushed pills?

**The children in this study were treated with crushed pills. Explained in lines 167-168.**

ii. a. Describe the prevalence and interventions of malaria in the study region, its typical yearly timing in relation to the baseline and follow up of this study.

**The prevalence of malaria and interventions that were conducted during the period that this study was carried out is described in lines 330 – 334.**

b. Is schistosomiasis liver fibrosis associated with liver image pattern C or B?

**In this study, liver image pattern C, indicating peripheral rings and prominent pipe stems is described and considered to be schistosomiasis-associated fibrosis. Lines 159 – 160.**

**Reviewer #2:**

i. While randomized, I don’t believe this can be considered a controlled trial since there is no control (untreated) group. Consider something like “a randomized dosing strategy trial” or “a randomized trial of one- vs. two-dose treatment”.

**The study title has changed to** **“Regression of *Schistosoma mansoni*-associated morbidity among Ugandan preschool children following praziquantel treatment: A randomised trial” with word controlled deleted since there was no control group in this study.**

ii. Clear the confusion in language with regard to claims about morbidity.

**The entire introduction corrected and rewritten. Lines 74 – 97.**

iii. Clarify the age groups that are being currently excluded from MDA in Uganda.

**Preschool children are maintained in the text; children who are young and not in school are not included in the national schistosomiasis MDA, in Uganda.**

iv. Without a control group the authors should have included uninfected children and followed for initial and final hepatomegaly.

**For ethical reasons it was not possible to have a control group of children that could not be treated lines 335-336. The children were their own controls as explained in lines 325-326.**

v. Please provide a citation for the use of a factor of 24 to convert eggs per slide to eggs per gram

**Citation for the use of a factor of 24 to convert eggs per slide to eggs per gram provided. Line 138 reference number 25.**

**Reviewer #3:**

i. Why did the authors not include measurements of portal vein dilatation and periportal vein thickening to identify schistosomiasis related morbidity?

**Measurements of portal vein dilatation and periportal vein thickening are not reported in this study because all children had normal sizes of hepatic portal vein at baseline and at follow-up. Lines 214–215.**

ii. Consider finding other references than the ones currently listed (4 and 5)

**References King 2006 and King 2010 deleted and relevant ones cited.**

iii. A short description of hemoglobin testing methods and estimation procedures should be included in the methods.

**Hemoglobin testing methods and estimation procedures included. Lines 139 – 144.**

iv. The reference for height-related data from a healthy Senegalese community should be put rightly.

**Reference corrected and changed. Lines 153-154 refence number 27, 28.**

v. Reference values for the liver and spleen measurements should be included in Table 2.

**Reference values for the liver and spleen measurements used in this study described in Table 2**

vi. Please give a detailed description of materials and methods for anaemia assessment and abdominal ultrasound examination.

**Fully described; lines 139 – 161.**

vii. Described the association of anemia with intensity of schistosomiasis infection.

**Heamoglobin level among children increased with decrease in intensity of *S. mansoni* infection. Discussed in line 300-303.**

**Reviewer #4:**

i. State whether this paper is a continuation of a previous clinical trial already published (PLoS Negl Trop Dis. 2015;9(5): e0003796).

**It is clearly stated in line 101-102 that this paper is a continuation of previous reports. References number 3, 9 and 17.**

ii. This study it should not be considered a randomized clinical trial.

**Although the study did not have a control group it remains a randomized trial as changed in the study title: “Regression of *Schistosoma mansoni*-associated morbidity among Ugandan preschool children following praziquantel treatment: A randomized trial”.**

iii. It should be stated in the methods that children received albendazole.

**It has been indicated that children received albendazole at baseline. Line 172-173.**

**Reviewer #5:**

i. The discussion needs thorough review of more literature.

**More literature has been cited in the discussion. Line 263 – 308.**

ii. It should be explained whether the study involved blinding.

**The nurses were blinded to the treatment groups. Lines 171.**

iii. **In Table 2: the p values are stated as ns if not significant at P > 0.05.**
iv. Discuss the schistosomiasis pathology in preschool children

***Schistosoma mansoni* related pathology discussed further. Lines 277-279.**

v. Were there any adverse effects following chemotherapy?

**There were no reports of adverse effects among the children following treatment with PZQ. Lines 177-178.**

vi. Discuss in detail the relationship between schistosome infection and anemia after treatment.

**The relationship between schistosome infection and anemia after treatment is discussed in detail. Lines 300-308.**
