# Supplementary material for: Regression of Schistosoma mansoni associated morbidity among Ugandan preschool children following praziquantel treatment: A randomised trial
Source: PLoS One. 2021 Nov 15;16(11):e0259338. doi: 10.1371/journal.pone.0259338 (PMC8592404; doi:10.1371/journal.pone.0259338)
Supplement: Attachment — Submitted filename: Response to Reviewers.docx [file pone.0259338.s004.docx]

**REBUTTAL LETTER**

David Joseph Diemert, M.D.
Academic Editor
PLOS ONE

Dear Sir,

RE: SUBMISSION OF MANUSCRIPT: **“Regression of *Schistosoma mansoni*-associated morbidity among Ugandan preschool children following praziquantel treatment: A randomized trial”** (PONE-D-20-12223R1).

We have carefully considered the academic editor and reviewer (s) comments and suggestions in the revised manuscript. As instructed, we have attempted to respond to all comments in point-by-point fashion.

Overall, the comments were very helpful and we are truly thankful for the constructive feedback on our original submission. We feel the quality of the manuscript is much improved after addressing the issues raised.

However, it was an arduous task trying to harmonize the sometimes-divergent views of all six reviewers. We did not receive a second set of comments from reviewer 3 and we assumed that reviewer 6 had not reviewed the initial manuscript.

Sincerely,

Allen Nalugwa

(Corresponding Author)

**Reviewer #1:**

The authors have responded to most of the reviewers' comments. However, the response to the question about malaria prevalence and interventions is not in lines 285-288 as the authors claim. Maybe the line numbers are off but this is of critical importance in considerations about anemia. They do semi-respond in the section on limitations of the study but overall, they could be more forthcoming about the possibility that the changes in anemia in the study participants could be confounded by malaria.

The issue about malaria are addressed in lines 288-290. The possibility of malaria influencing indices is acknowledged and discussed in the manuscript (see limitations section) as well as discussion lines (297, 319-323).

Other minor corrections:

Line 79 should be a less than or equal to sign rather than a greater than or equal to sign.

The correct sign has been added line 74.

Line 131 is only the magnification of the objective lens, when coupled with the ocular lenses, the overall magnification is 100X. This has been corrected line 128.

Line 211 says children who had liver pathology (defined by the authors as Image Pattern C) were more likely to be lost to follow up. This is not true as the p value is 0.76.

The misleading statement has been deleted.

**Reviewer #2:**

General: In some cases, authors have responded satisfactorily to my initial comments, however, the point-by-point reply was a highly compressed summary in which many comments were not addressed, and the manuscript revisions also have not addressed them. I have copied these from my original review below, with slight editing in some cases.

**Introduction**: Currently, authors are primarily using the introduction to make the case that preschool-aged children should be treated at all, but then the study focuses on frequency of treatment instead. We need additional text to make the case to the reader of why looking at 2 vs 1 dose adds value. I.e., was there data or concern that younger kids were likely to require a double dose and thus would not benefit from inclusion in MDAs (which to my knowledge are only single-dose)?

We do not understand this argument. The study does not focus on the frequency of treatment. It compares morbidity regression regarding use of treatment with one or two doses. Frequency would relate to whether MDA treatment should for example be annual or every two years or once before children start school. The issue of multiple dosing to improve cure rates of infections attributable to *S. mansoni* is addressed in reference 17.

Methods: not necessarily a weakness, but I’m curious as to why authors did not choose to collect a followup stool sample to test the impact of treatment on stool positivity or epg. Would briefly address your reasoning here or in the Discussion.

As stated, this report relates to long term morbidity. Follow-up stool samples to test for egg positivity were done to assess cure rate at six weeks and these results are reported elsewhere in reference 17.

Methods: Similarly, I’m curious as to why authors assessed stool egg density on enrollment, but chose not to do anything with that data except descriptive analysis comparing followed children with those lost to followup. To me, the obvious next step would have been to stratify children by initial infection density when analyzing for hepatomegaly regression with treatment. And given the uncontrolled nature of the study, a pattern showing more improvement in more heavily infected children could help bolster your case that the improvement seen did in fact result from treatment. Or if authors intend to publish this separately instead, would just say so.

The study was not designed and powered to perform this stratified analysis. The intensity of infection was associated with level of hepatomegaly and these results are already published reference 9.

In the discussion: What are the implications of the above issues [around logistical challenges of reaching out-of-school children] for your recommendations…?

We do not understand this comment However, we state in the study limitations that one dose is more likely to logically reduce logistical challenges. Line 308-309

Finally, a few new comments based on the revised Discussion text:
Discussion: Please strengthen your argument by briefly assessing alternative explanations for the improvements in hepatomegaly seen with treatment. To me the most obvious would be if malaria was responsible for some of the initial hepatomegaly, and local seasonality patterns are such that community infection prevalence is lower around the time of the followup testing. (The brief mention of malaria seasonality does not address this.)

We discuss this issue by stating and contextualising the seasonality of malaria, perennial transmission and high coverage with insecticide treated nets (ITNS). Lines 297, 319-323. The direction of malaria could actually act in both directions to increase or decrease level of hepatomegaly. Malaria would also probably change the size of the spleen as well as both lobes of the liver. We compare changes in spleen sizes as well as in both lobes of the liver to put our findings into wider perspective.

Another possibility which is important in some SSA regions is aflatoxin exposure depending on the local staple food and storage practices.

The issue of aflatoxin and other liver toxins/injuries are clearly beyond the scope of the current study.

Discussion line 269: this is the first reference I’ve seen to a cure rate being determined in your study. If these children were in fact followed up with repeat stool testing, at minimum a brief mention of the findings and a reference to where they are more fully presented is needed.

We do not understand this statement. The current study is not about cure rate but about morbidity regression. In schistosomiasis this can be confusing. Cure relates to clearing of infection (as measured by reduction and or cessation in egg production) whereas morbidity regression relates to residual effects of infection such as hepatomegaly and or hepatic fibrosis. The results of cure are already reported in reference 17.

Discussion lines 277-281: It appears you are largely basing the argument that treatment earlier in childhood may decrease clinical morbidity (liver fibrosis and failure) in adulthood on the full resolution seen in your few cases diagnosed with periportal fibrosis. 1) are you sure that it’s appropriate to interpret these sonographic findings in children using fibrosis criteria developed for adults, when the reversibility itself may suggest they were not true fibrosis? 2) to make this argument you would need to briefly provide comparisons – did the other studies in children show the same findings? In contrast, have studies in adults shown that these findings do not resolve with treatment?

1. We just make a hypothesis given the limitations of this study (few children with hepatic fibrosis).
2. The sonographic findings used are applicable to older children as well as adults. References 23 and 24.
3. References 34 and 36 are included to compare with older children.
4. As to whether findings do not resolve with treatment. This question is beyond the scope of the current study and may be a subject of further inquiry (more studies).

**Reviewer #4:**

Unfortunately, my comments were not addressed

All the comments were responded to. We have copied and pasted them here and given point by point responses.

The paper seems to be continuation of a previous clinical trial already published (PLoS Negl Trop Dis. 2015;9(5): e0003796), so that point should be stated clearly somewhere.

This is acknowledged in the methods see line 96-97.

Through both papers it is quite clear that beyond those studies there is a big logistical investment for carrying such research work on the “field”.

Clear, this is what we are demonstrating in this manuscript.

My major concern relies on the design of the study. The fact of that work be the continuation of a previous trial is because methodologically is crucial. Authors refers to the sample size calculation to the previous study, where the number of participants were estimated according to another different outcome (cure), so what authors are doing is following the previous cohorts. Therefore, strictly it is not a randomized clinical trial.

This can be a grey area. We use a wide NIH definition of a clinical trial “a research study in which one or more human subjects are prospectively assigned to one or interventions. See [www.grants.nih.gov](http://www.grants.nih.gov). We are following two groups randomised to either single or double dose.

That could have an important impact in the measure of the intervention effect (mainly due to the lower expected numbers of patients who complete the follow up period).

We performed a power analysis because of this limitation and the power was adequate (see page 13 lines 312-317 we also discuss this limitation.

On the methods section, authors say that stool parasitological stool examination would be performed, but there are no results regarding that issue.

This issue has been raised by reviewer 2. These results are addressed and published in reference 17.

One of the variables that are measured is hemoglobin. Analyzing the results, probably, is one of the parameters that have been more affected by the intervention. Unfortunately for the study, it is stated in the discussion section that patients received albendazole. That information should be added at the methods.

The issue of Albendazole is addressed in the methods section (line 169-170 page 7)

Considering those facts what it could be said is that authors could not demonstrate if there is any difference between one or two doses, basically because the study was not designed for that. Agreed, but we have enough power > 80%.

Neither can it be said that: “…any of doses of praziquantel are effective in reversing morbidity attributable to *S. mansoni*” What it has been improved are only a few of the several parameters identified by authors as related to the effect of S. mansoni (being one of them the Hb, but confounded with the effect of albendazol)

- We attenuate our conclusions given the limitations see discussion lines 259-268.
- The albendazole is discussed in lines 294-296.
- These parameters are actually measures of *S. mansoni* associated morbidity

In summary my opinion is that the paper could be published but need to be rewritten taking into account the important methodological limitations and being more realistic when obtaining conclusions.

As a final minor comment, table 3 should be expressed as table 2 (same columns….)

This was corrected (line 209)

**Reviewer #5:**

Not all reviewers' comments have been answered. The lack of a control group cannot be explained. The children cannot be their own controls, as we cannot know how the natural evolution of a hepatomegaly would be, for example, in a non-infected child, since we cannot rule out the possibility that it may resolve spontaneously, be related to the seasonality of malaria, other conditions, etc. Therefore, this has to be clearly specified as a major limitation of the study. Although the section on limitations contains these considerations, it immediately considers them as not relevant, when they should be considered as possible and relevant biases.

We agree that children cannot be their own controls in this context.

We have changed this. The limitations are now better written lines 320-321.

The text specifies that the treatment was blind for the nurses, but it does not explain how this was done, a placebo was used for the second dose? How was it adressed at that time?

This has now been corrected no placebo was given. See line 167-168.

The limitation on the absence of results of parasitological control and the intensity of the infection has not been answered.

This has been addressed. Children were tested for cure at 6 weeks and results are reported elsewhere (reference 17). There are not enough numbers for stratified analysis depending on intensity of infection.

**Reviewer #6:**

Abstract: Line 52 - you probably mean that you took haemoglobin measurements, as 'anaemia' cannot really be measured directly.

**Sure, we have replaced anaemia with heamoglobin line 52.**

Line 55 - you probably mean that there was no difference between the changes in proportion of children with enlarged liver, because that is what your outcome measure is; it is not 'liver size' per se as implied by this sentence. In both line 53 and 54 you should include an estimate of the differences between those two pairs of proportions with the 95% confidence intervals (and p-values if you wish). Same in line 57 and line 60. Line 60 - the conclusion that these results suggests that one dose is as effective as two doses is incorrect given the study design (that would have been correct if this was a non-inferiority trial); the more appropriate conclusion is that there is no evidence of a difference in effect between the two doses. **Corrected. Lines 53-63**

**Introduction:**
Line 84 - check that you really mean that the tablet is 'sour' (like lime) and not 'bitter' (like quinine). You refer to bitterness in your response to the previous round of reviews and also in line 170. This has been corrected line 79 and 167.

**Methods:**
Line 113 - what you mean is the 5% level of significance, not the 95% level.

Changed, line 110.

Also, this description of the sample size calculation is incomplete, as it should explicitly state the outcome upon which it was based (morbidity is generic) and also state the proportion of this outcome in at least one treatment group (usually the comparison/baseline/control group).

This has been taken care of see lines 106-112.

The McNemar-Bowker test is not appropriate for these data because individuals in these data are not paired.

We have a problem with this assertion! The McNemar-Bowker test is used for *matched pairs*. Such pairs may involve 1) repeated measures (e.g., pre treatment-post treatment design), 2) matched pairs involving treatment and control subjects, 3) within-subjects’ experiments (e.g., participants receive both treatment and control) or 4) multiple dependent measures for a respondent.

See references for utility of this test.

1. Bowker, A. H. (1948). A test for symmetry in contingency tables. *Journal of the american statistical association*, *43*(244), 572-574.
2. Cochran, W. G. The comparison of percentages in matched samples. *Biometrika*37256–266
3. Krampe, A., & Kuhnt, S. (2007). Bowker's test for symmetry and modifications within the algebraic framework. *Computational statistics & data*

We did not use the MCNemar-Bowker test to compare morbidity regression between one dose and two doses. We instead compared for a change in parameters before treatment and at follow up within the two groups using the Chi-square test statistic with Yates correction.

You should instead conduct a regression analysis such as binomial regression to estimate proportion (risk) differences with their confidence intervals (e.g. 'binreg' in Stata with the 'rd' option for risk differences). **See comments above regarding this!**

Furthermore, a comparison of baseline characteristics between groups is not appropriate for a randomised group assignment as the groups are expected to be balanced and any observed differences are likely to be due to chance.

**Sure? It however may show whether the randomisation was balanced!**

Results:
Table 1 should describe the baseline characteristics of the children retained in the study only, comparing between those assigned to the two doses. However, there should be no statistical tests comparing the groups. Done, see table 1. Lines 201-202

The current Table 1 is also useful as-is, but should be moved to an appendix, with a comment in the main text (e.g. what's currently stated in lines 202 to 203) describing how the retained sample and the lost-to-followup groups compare. The focus of the first paragraph should be on describing the retained sample as shown in this new table 1 which I am suggesting to be included. In this table counts and proportions of categorical variables, and means and SDs or medians and IQRs of continuous variables should be reported - remember, no statistical tests comparing the two dosage arms.

- Done see lines 203-204.
- Appendix/Supporting Information S5, line 333.
- Table one changed as suggested.

However, this guidance is at variance with that of reviewer one. We have decided to take it, as it does not change the quality of the manuscript.

The description of mordibity in line 213 belongs to the methods.

Sure, it is repeated here for emphasis.

The analysis and presentation of results in table 2 is inappropriate. I would advice that you define the outcomes as binary variables (i.e. enlarged vs not) and then fit a model estimating risk differences for enlargement pre-vs-post in the two treatment groups. To do this you would first format the data such that each row represents one observation per child per timepoint, meaning each child would appear twice in the data: once for the pre- and once for the post- treatment times. You would need a variable indicating timepoint (e.g. 0 for pre-treatment and 1 for post-treatment). You would also have a variable for treatment group i.e. single vs double dose. Then fit a model with each binary outcome and with interaction between treatment group; you can subsequently include covariates if you also want adjusted estimates. For example, in Stata: binreg outcome treatment_group##time, rd (you can then repeat this with any adjusting variables). In this model, the effect of the treatment_group#time interaction, estimated as a risk difference, is the difference in pre-vs-post treatment risks of organ enlargement between the groups. This is what you should then report for each outcome in Table 2 as the treatment effect with 95% confidence interval and p-value. You may report both unadjusted and adjusted effects. Please consult a statistician for advice on this. You can do the same for anaemia (as a binary variable) or fit a linear regression model with the same interaction above with continuous haemoglobin. Each results table should include the count and proportion of the outcome pre-treatment and post-treatment in each arm (or the mean and SE for continuous outcomes) and the treatment effect estimated as I have described, with the 95% CI and p-value.

Thank you for these observations. These suggestions were actually pondered on earlier but not used for several reasons.

1. We wish to compare morbidity regression of one versus two doses. The suggested pooled analysis blurr that aspect.
2. Lumping schistosomiasis related morbidity as present or absent is problematic. Liver size and haemoglobin levels present varying spectrum (normal, moderate, severe) rather than a dichotomy. We would wish to retain this level of spectrum in our data as well as in our analyses.
3. Some children may move from severe to moderate and this is an improvement. Dichotomization of outcome would not be sensitive enough to capture this change.
4. The aim of the study is not to identify risk factors for morbidity regression.

The organ sizes reported here are actually standard deviations not original sizes (see references 27 and 28). We are hesitant to do further statistical manipulation of these variables because of danger of having analytical errors due to ignoring context. See paper by Miguel A Herna, David Clayton and Niels Keiding. The Simpson’s paradox unraveled, International Journal of Epidemiology 2011;40:780–785 doi:10.1093/ije/dyr041.
