# Supplementary material for: Regression of Schistosoma mansoni associated morbidity among Ugandan preschool children following praziquantel treatment: A randomised trial
Source: PLoS One. 2021 Nov 15;16(11):e0259338. doi: 10.1371/journal.pone.0259338 (PMC8592404; doi:10.1371/journal.pone.0259338)
Supplement: Attachment — Submitted filename: Response to Reviewers.docx [file pone.0259338.s005.docx]

**REBUTTAL LETTER**

David Joseph Diemert, M.D.
Academic Editor
PLOS ONE

Dear Sir,

RE: SUBMISSION OF MANUSCRIPT: **“Regression of *Schistosoma mansoni*-associated morbidity among Ugandan preschool children following praziquantel treatment: A randomized trial”** (PONE-D-20-12223R2).

We have carefully considered the reviewer(s) comments and suggestions in the revised manuscript. As instructed, we have attempted to respond to all comments in point-by-point fashion.

We feel the quality of the manuscript is much improved after addressing the few issues raised.

Thank you for the helpful comments!

Sincerely,

Allen Nalugwa

(Corresponding Author)

02/05/2021

**Reviewer #2:**

**Introduction:**

This is a restatement of the comment on the Introduction in my last review which reviewers noted they did not understand. There is still a crucial disconnect between the way the authors frame the study’s contributions and what the study design can actually show. Most of the introduction is about the exclusion of PSAC from praziquantel MDAs altogether, and the authors situate their findings as “helpful in guiding policy and practice decisions regarding the routine treatment of this important age group”. But then the study design looks at morbidity regression with 1 vs 2 doses. Demonstrating that children getting 1 dose had equivalent medium-term improvement those getting to 2 does not demonstrate that treatment caused those outcomes and is superior to no treatment. Though this issue risks causing important confusion to readers, it may be a purely semantic problem that could be easily addressed by reframing to state that this study was intended to determine two things: 1) whether morbidities regressed with treatment at all over time, and 2) if so, whether one dose was equivalent to two since it would be simpler to achieve. Currently the introduction conflates these two concepts.

Changed as advised, lines 90-92.

**Discussion:**

The authors continue to frame the results as clearly showing that PZQ treatment was effective in causing the morbidity reductions seen in their study. The study design does not allow making this determination and findings should not be described as representing effectiveness at all. The authors have rejected suggestions in previous comments to nuance this claim by providing alternate explanations for the changes seen (e.g. malaria – which is discussed extensively as an explanation for findings from other studies, but not as a possible cause of spurious association with treatment for their own findings.) But even these comments would only have helped in the context of a larger reframing that has not been done: acknowledging that an uncontrolled pre-post study cannot prove causality, and at best can only suggest an association worth investigating further. Without an uninfected (not untreated) control group followed on the same outcomes over the same time period, we cannot rule out changes in malaria prevalence, other exposure, or even natural changes with age, as the cause of improvement in findings. (E.g. is organomegaly more prevalent in younger children in general than older ones in this population?) (The Kenyan schistosomiasis study referenced in the reply also followed an uninfected comparison group over time.) Fundamentally, the claims for the type and strength of evidence this provides need to be dialed back and the authors need to proactively point out the remaining evidence gaps. But authors can still justly claim that their study provides longer-term follow-up data than was previously available in PSAC and will help guide dosing if/when programs begin to target PSAC.

We hope that we understand the arguments this time around.

1. The results of this study cannot be interpreted in isolation. PZQ is known to modify hepatic pathology as provided in references 33-36 within the write-up. However, we discuss other causes of the pathology and put them in context within this study. See references 37-42.
2. We also state the ethical dilemma of not to treating some children as controls.
3. Uninfected children in Kenya study cannot be a control group. Results of follow-up for the uninfected group are different from the infected children (reference 7).

**Reviewer #5:**

Most of the issues have now been addressed and corrected. I think the article is suitable for publication, I have two minor comments at the moment.

**Line 317**: “Consequently, our results should be 318 generalizable to children less than six years infected with S. mansoni”. This statement is difficult to maintain in a limitations section, without a control group.

Statement deleted. Page line 316-317.

**Line 322:** “Besides, the major control strategy for malaria in the study area is with use of insecticide treated mosquito nets with a very high household coverage of > 90%”. In the same way, this statement is not justified in a limitation section.

Statement deleted. Page line 320-322.

**Reviewer #6:**

**Major comments:**

The authors have presented a rebuttal to my previous suggestion of the appropriate approach to analysis of these data. I am not convinced by the rebuttal for the following reasons:

(1) The study compared a single vs. double dose of praziquantel on its effect on regression of morbidity as measured by the sizes of their spleens and livers as well as liver fibrosis. The authors cite the proportions with spleen enlargement, liver enlargement and liver fibrosis (all binary outcomes) in the two treatment groups but yet in the methods and in Table 2, these outcomes are presented as three-level categorical variables. Perhaps this was not the authors original intention and this has been introduced during peer review. However, one reason why I am not convinced by the authors rebuttal that they were interested in liver size as a 3-level spectrum is that the sample size calculation is based on a binary outcome! The way an outcome is specified for the sample size is usually the way it is specified in the main analysis. In any case, it would have been statistically even more powerful to use the actual liver/spleen size e.g. in centimeters as the outcome of the analysis rather than categorizing it to 3-levels, if the worry is about blurring that aspect of morbidity as the authors indicate. Alternatively, there is the option to use an ordinal logistic regression to model the 3-level ordered categorical outcome and adjusting for the baseline level of the outcome, with the treatment indicator as the main explanatory variable.

Morbidity regression is assessed by size of the liver which is not a binary outcome. As stated previously the three levels come by re-categorizing liver size according to standard deviations from the mean (references 27 and 28). The organ sizes reported here are actually standard deviations not original sizes (see references 27 and 28). We are thus hesitant to do further statistical manipulation of these variables because of danger of having analytical errors due to ignoring this peculiar context related to Schistosomiasis morbidity. See paper by Miguel A Herna, David Clayton and Niels Keiding. The Simpson’s paradox unraveled, International Journal of Epidemiology 2011;40:780–785 doi:10.1093/ije/dyr041

(2) assuming the outcomes are in fact 3-level categorical variables, in table 2, you present p-values comparing each level of each 3-level categorical variable in the pre- vs post- treatment period, repeating this for the single dose and double dose arms. This is inappropriate because firstly, these three categories are not independent of each other, since belonging to one category necessarily means you don't belong to the other two categories. Secondly, comparing the pre- and post-treatment groups in each of the arms separately does not tell us anything about how the arms compare to each other, which is the aim of the study! These p-values are therefore meaningless in as far as evaluating evidence for the difference between the two treatments is concerned. The P-values have been corrected (Table 2)

The authors assert that McNemar-Bowker test is appropriate in this setting because observations are paired in the sense that there are pre-treatment and post-treatment in the same individuals. This is a misinterpretation of the concept of pairing in as far as exploring the causal effect of treatment is concerned. In this setting a pre-treatment value of an outcome is a 'baseline' and has not been affected by treatment; it is a value to be adjusted for in the analysis. Only the post-treatment values of the outcome are relevant for the comparison of the treatment groups as they are the only values expected to have been causally influenced by treatment. Hence my previous suggestion to fit a model which estimates the difference in the post-versus-pre-treatment values of the outcome in the one dose versus two dose groups i.e. a difference-in-difference analysis (or alternatively as discussed above linear regression on liver and splenic sizes in cm and even heamoglobin post treatment, or ordinal logistic regression on 3-level categorical measures, adjusting for the pre-treatment values with the one vs. two dose variable as the main explanatory variable, with or without adjustment for other factors so desired).

We detected and analysed for a change between follow up and baseline variables not merely baseline and or follow-up. We still have a problem with the current assertion! The McNemar-Bowker test is used for *matched pairs*. Such pairs may involve 1) repeated measures (e.g., pre treatment-post treatment design), 2) matched pairs involving treatment and control subjects, 3) within-subjects’ experiments (e.g., participants receive both treatment and control) or 4) multiple dependent measures for a respondent. We did not use the McNemar-Bowker test to compare morbidity regression between one dose and two doses. We instead compared for a change in parameters before treatment and at follow up within the two groups using the Chi-square test statistic with Yates correction.

This is a good study and I think that if the authors could improve the statistical analysis as advised, perhaps consulting with a statistician as well, I think it would be a great paper. The authors should also look at the CONSORT guidelines to guide them on how and what to report in each section of the manuscript. I sympathize with the authors having to deal with multiple review comments, and as far as I can see, there is no contradiction in reviewers' feedback as far as the statistical analysis and reporting is concerned. Five out of six reviewers state that statistical analyses been performed appropriately and rigorously.

**Minor comments:**

In the response document you indicate that you have changed the description of the pill from 'sour' to 'bitter' but the text still says sour. Corrected, line 79.

I had suggested previously that the difference-in-difference of the pre- vs post-treatment prevalence in the two groups be presented, with confidence intervals (and p-values if desired) but this has not been done. Without this, it is not possible to evaluate the conclusion that there is no evidence of a difference in effect - the reader needs to see this difference in effect with its confidence intervals and note whether it does or does not exclude no difference. I also note that parameters for a sample size calculation are now presented i.e. assuming a difference of at least 12% and assuming 75% prevalence of the primary outcome in the two-dose group. It is not enough to present these parameters without justification e.g. some citation showing that these would be the expected values or why a 12% difference is clinically important. The 'sample size' section should be moved to just above the statistical analysis section.

The 'sample size' section has been moved to just above the statistical analysis section, lines 168-175.
