# Supplementary material for: Regression of Schistosoma mansoni associated morbidity among Ugandan preschool children following praziquantel treatment: A randomised trial
Source: PLoS One. 2021 Nov 15;16(11):e0259338. doi: 10.1371/journal.pone.0259338 (PMC8592404; doi:10.1371/journal.pone.0259338)
Supplement: Attachment — Submitted filename: Response to Reviewers.docx [file pone.0259338.s006.docx]

**REBUTTAL LETTER**

David Joseph Diemert, M.D.	03/06/2021
Academic Editor
PLOS ONE

Dear Sir,

RE: SUBMISSION OF MANUSCRIPT: **“Regression of *Schistosoma mansoni*-associated morbidity among Ugandan preschool children following praziquantel treatment: A randomized trial”** (PONE-D-20-12223R4).

We have carefully considered both the editor and reviewer’s comments and as instructed; we have attempted to respond to all comments in point-by-point fashion.

The changes made have been marked in the ‘Revised Manuscript with Track Changes'.

We feel the quality of the manuscript has greatly improved after attending to the issues raised.

Thank you for the useful observations and helpful comments!

Sincerely,

Allen Nalugwa

(Corresponding Author)

**Editor comment**

**Please upload a copy of Supporting Information Figure S2 which you refer to in your text on page 14.**

**Figure 1 was misplaced under Supporting Information as Figure S2 Fig 1 tiff and it has been deleted. Line 323 page 14**

**Figure 1 has been reuploaded under figure file.**

**Reviewer #2:**

**I am baffled as to why the authors are repeatedly refusing to state consistently and clearly in the manuscript a crucial interpretation point that they appear to agree with in their response letters: An uncontrolled pre-post study design cannot prove causality.**

**We have had a big problem with these assertions.**

**1. The aim of this study is not to establish/prove causality. Causality is already known.**

**2. The data ought to be interpreted against this body of knowledge and not in isolation.**

**3. It is unethical to have a control group that cannot be treated!!!**

**Listing possible alternate explanations in the Discussion does not compensate for repeatedly and inappropriately using words like "effectiveness" and "effect" instead of "association".**

**See comments above. Although it does not compensate it puts the study in perspective signaling that data should be interpreted with caution. However, by casting the study in available literature and known knowledge there is a higher likelihood that the observed changes are “causal”. Inconceivable that one can treat schistosomiasis to observe an “association”.**

**There is even the claim that MDA policy should be altered based on their findings, when the type of data presented does not meet the standard of evidence used for e.g. WHO policy recommendations. This is not a criticism of the study or the data, which in fact is valuable and should be considered within the body of evidence in policy updates.**

**The study adds to the body of knowledge suggesting that there is need for a change in policy. If children improve after treatment, they should be treated!**

**The meaning of the rebuttal claim that comparing evolution of ultrasound abnormalities over time in infected treated vs uninfected PSAC cannot b done because 'results are different' is also unclear. However, even if there is a biomedical reason this comparison would be unhelpful, there remains the basic problem: we don't know whether the abnormalities initially seen in the enrolled children are 'worse' than would have been seen in uninfected children, or if the improvement over an 8-month period would have happened regardless of treatment. None of this is a problem with the study itself, but with the claims being made in the Discussion about what the study can prove.**

**We think this came from a previous comment about the Kenyan study. This issue was actually raised by the reviewer previously (second review) (reference 6).**
